# Time-course of sFlt-1 and VEGF-A release in neutropenic patients with sepsis and septic shock: a prospective study

**DOI:** 10.1186/1479-5876-9-23

**Published:** 2011-03-03

**Authors:** Brunna E Alves, Silmara AL Montalvao, Francisco JP Aranha, Irene Lorand-Metze, Carmino A De Souza, Joyce M Annichino-Bizzacchi, Erich V De Paula

**Affiliations:** 1 Hematology and Hemotherapy Center, University of Campinas, Campinas, SP, Brazil; 2 Faculty of Medical Sciences, University of Campinas, Campinas, SP, Brazil

## Abstract

**Background:**

Septic shock is the most feared complication of chemotherapy-induced febrile neutropenia. So far, there are no robust biomarkers that can stratify patients to the risk of sepsis complications. The VEGF-A axis is involved in the control of microvascular permeability and has been involved in the pathogenesis of conditions associated with endothelial barrier disruption such as sepsis. sFlt-1 is a soluble variant of the VEGF-A receptor VEGFR-1 that acts as a decoy receptor down-regulating the effects of VEGF-A. In animal models of sepsis, sFlt-1 was capable to block the barrier-breaking negative effects of VEGF-A and to significantly decrease mortality. In non-neutropenic patients, sFlt-1 has been shown to be a promising biomarker for sepsis severity.

**Methods:**

We prospectively evaluated concentrations of sFlt-1 and VEGF-A at different time-points during febrile neutropenia, and evaluated the association of these levels with sepsis severity and septic shock development.

**Results:**

Neutropenic patients that evolved with septic shock (n = 10) presented higher levels of sFlt-1 and VEGF-A measured 48 hours after fever onset than patients with non-complicated sepsis (n = 31) and levels of these biomarkers correlated with sepsis severity scores. Estimation of the diagnostic accuracy of sFlt-1 levels for the discrimination of patients that evolved to septic shock yielded promising results in our study population.

**Discussion:**

Our data suggest that sFlt-1 and VEGF-A could be useful biomarkers for sepsis severity in patients with febrile neutropenia. In addition, the kinetics of sFlt-1 release in patients that evolve to septic shock suggest that the sFlt-1 could be a salvage compensatory mechanism in patients with septic shock, but that the magnitude of the sFlt-1 release observed in human sepsis is not sufficient to reproduce the beneficial anti-VEGF-A effects observed in animal models of sepsis.

## Background

Patients with hematological malignancies submitted to intensive chemotherapy present a higher risk of sepsis and sepsis complications. Febrile neutropenia (FN) in these patients is considered a medical emergency, and a standardized management approach including wide-spectrum antibiotics and admission is usually implemented for all patients. So far, there are no reliable laboratory markers to indicate whether FN patients will recover uneventfully or rapidly deteriorate to sepsis, septic shock and death [1,2].

Vascular endothelial growth factor (VEGF-A) is an endothelial growth factor that is widely known for its key role in the regulation of embryonic and post-natal angiogenesis. However, VEGF-A was first characterized by its endothelial barrier-breaking properties, as a potent stimulator of endothelial permeability [3]. This capability to disrupt the integrity of an endothelial cell tube is in fact very important during angiogenesis, as new cells have to be incorporated in a growing vessel. Recently, this property has been explored as a putative common downstream mechanism in pathological conditions associated with loss of endothelial barrier function. In line with this hypothesis, several authors have demonstrated elevated VEGF-A levels in intensive care units (ICU) patients with sepsis, as well as associations between VEGF-A levels and sepsis severity [4-6].

sFlt-1 is a natural splice variant of the tyrosine-kinase receptor Flt-1, which is an endothelial cell receptor for VEGF-A. sFlt-1 binds free VEGF-A and acts as its antagonist [7]. In animal models of sepsis, sFlt-1 has been shown to attenuate the severity of the inflammatory response and to antagonize the barrier-breaking properties of VEGF-A, thus suggesting a therapeutic role for this protein [8]. The potential value of sFlt-1 as a biomarker for sepsis severity has been demonstrated in studies with non-neutropenic patients [6,9].

Here we prospectively evaluated the serial expression of sFlt-1 and VEGF-A in patients with hematological malignancies and chemotherapy-related FN, to gain insights about both potential roles of sFlt-1 in patients with febrile neutropenia and sepsis, as a biomarker or as a therapeutic tool.

## Methods

### Patient's eligibility criteria

Recruitment took place at the Bone Marrow Transplantation Unit of our University hospital between March 2008 and March 2009. Inclusion criteria were: (1) diagnosis of hematological malignancies, and (2) admission as inpatients for intensive chemotherapy (induction for acute leukemia or high-dose sequential therapy for lymphomas) or hematopoietic stem-cell transplantation (HSCT). Patients were invited to participate before the initiation of chemotherapy. The study was performed in accordance with the Declaration of Helsinki and approved by the local ethics committee and informed written consent was obtained from all patients. Fever (T≥38.0°C) at admission for chemotherapy was the only exclusion criteria, but only patients that presented fever during neutropenia (defined as a neutrophil count <500⁄μl) were included in the second phase (see laboratory measurements). Clinical data were obtained from the medical records.

### Sepsis definitions and risk stratification scores

Sepsis, in this population, was defined by the presence of two or more of the following: (1) temperature > 38.0°C, (2) heart rate > 90 beats/min, (3) respiratory rate > 20 breaths/min or PaCO_2 _< 32 mmHg; and a microbiologically proven or clinically evident source of infection [10]. In accordance with current management protocols, an infectious etiology was assumed for all FN patients, and broad-spectrum antibiotics were initiated immediately after cultures were obtained [11]. Septic shock was present in patients in which sepsis was complicated with hypoperfusion or hypotension (systolic arterial pressure <90 mmHg or a reduction in systolic blood pressure of >40 mmHg from baseline), despite adequate volume resuscitation. Severity of illness was assessed by calculating the Sequential Organ Failure Assessment (SOFA) score [12] daily after the development of fever. Patients were also stratified by the Multinational Association for Supportive Care In Cancer (MASCC) score at the time of fever [13,14]

### Laboratory measurements

Venous blood was drawn within 12 hours after first episode of neutropenic fever, and 48 hours thereafter. Serum levels of VEGF-A and sFlt-1 were measured in duplicate using a commercial enzyme-linked immunosorbent assay (ELISA) kit (Quantikine, R&D Systems, Minneapolis, MN, USA) according to the manufacturer's instructions.

### Statistical Analysis

Patients were divided in two outcome subgroups according to the presence of absence of septic shock at any time point before the resolution of neutropenia and before 30 days. Differences in continuous and categorical variables were analyzed using the Mann-Whitney or Fisher's exact test respectively. Data are expressed as median and range unless otherwise stated. Correlation analysis (Spearman's rank correlation) was performed between sepsis severity scores and VEGF-A e sFlt-1 concentrations. Receiver operator characteristics (ROC) procedures were used to estimate diagnostic accuracy. A P value less than or equal to 0.05 was considered statistically significant. All statistical analyses were performed with the GraphPad Prism Software (GraphPad Prism Software Inc. San Diego, California, USA).

## Results

### Patients Characteristics

**Figure 1:**
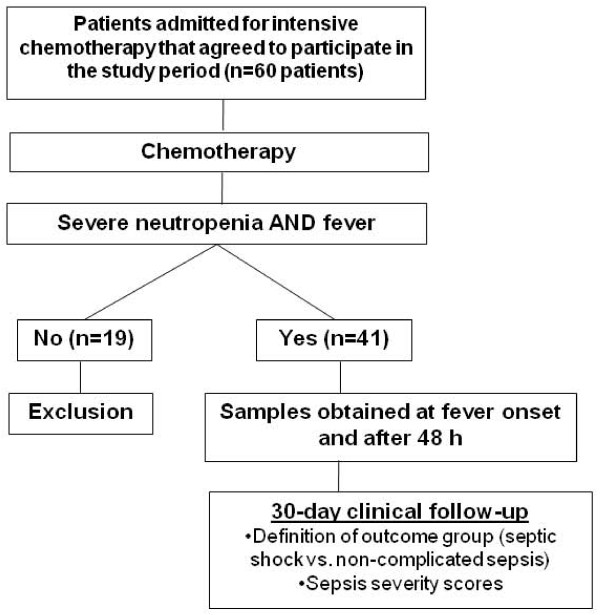
**Study flowchart**.

Of 60 patients that were included in the study, only 41 experienced neutropenic fever and completed the study (Figure 1). Patient characteristics are shown in Table 1. Septic shock during the period of neutropenia requiring mechanical ventilation was present in 10 patients, but was not present at study entry in any of the patients. Median time to septic shock development was 4.1 days (range 1 - 7 days) after the first episode of neutropenic fever, and in only one patient, septic shock onset occurred in the first 48 hours after study entry. The median time between the onset of septic shock and the need for mechanical ventilation was 1 day (range 0-2 days). Eight patients died from complications of sepsis within the first 30 days after the onset of fever, yielding an overall 30-day mortality of 13.3%. Clinical significant differences between patients with non-complicated sepsis and septic shock included age, presence of bloodstream infection, SOFA score 48 hours after fever onset and MASCC score at FN onset. Gram-negative and Gram-positive organisms were isolated in 7 and 6 patients respectively, whereas fungi were isolated in 3 patients. Isolated microorganisms included: A. baumannii, E. coli, K. pneumoniae, P. aeruginosa, E. cloacae, S. aureus, S. epidermidis, S. viridans, Fusarium and Aspergillus. Four patients had blood cultures positives for two pathogens.

**Table 1:** Patient characteristics

	**Sepsis **¥ (n = 31)	Septic shock (n = 10)	P
**Gender**			0.16 **
Male	13 (42%)	7 (70%)	
Female	18 (58%)	3 (30%)	

Age (median, range)	37 (16-55)	55 (24-62)	P < 0.01 *

**Disease status**			0.12 **
Complete remission	13 (42%)	1 (10%)	
Active disease	18 (58%)	9 (90%)	

**Treatment**			0.48 **
Intensive CTx (includes autologous HSCT)	17 (55%)	6 (60%)	
Allogeneic HSCT	14 (45%)	4 (40%)	

**Neutrophils/μl - Fever **(median, range)	60 (0 - 290)	50 (20 - 470)	0.40 *

**Days of neutropenia **(median, range)	12 (4 - 22)	14 (7 - 30)	0.36 *

**Platelets ×10^3^/μl - fever **(median, range)	25 (6 - 169)	38 (12 - 90)	0.16 *

**Days with fever **(median, range)	4 (1 - 12)	5 (1 - 12)	0.65 *

**SOFA score - fever onset **(median, range)	3 (0 - 7)	4 (2 - 8)	0.31 *

**SOFA score - 48 hours **(median, range)	4 (2 - 7)	7 (4 - 16)	P = 0.01 *

**MASCC score **(median, range)	21 (16 - 23)	18 (11 - 24)	P = 0.03 *

**Agent isolation in bloodstream**			P < 0.001 **
Yes	4 (13%)	8 (80%)	
No	27 (87%)	2 (20%)	

¥ Non-complicated sepsis; * Mann-Whitney test; ** Fisher's exact test. HSCT: Hematopoietic stem cell transplantation; CTx: chemotherapy.

### Time-course of sFlt-1 and VEGF-A expression in FN

**Figure 2:**
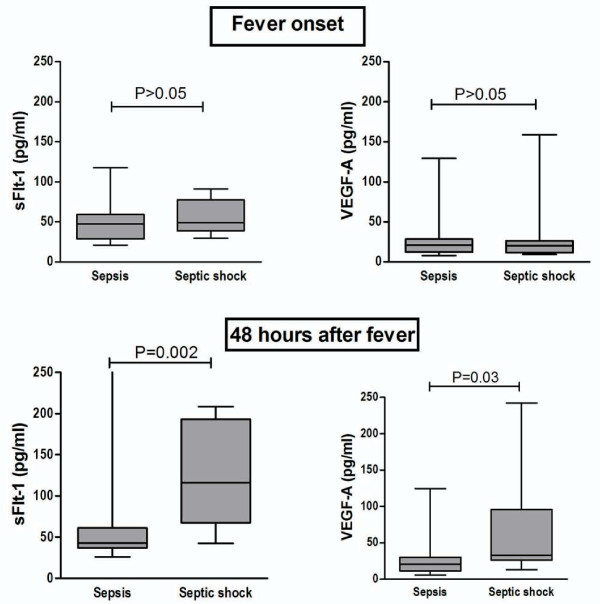
**Serum sFlt-1 and VEGF-A levels in FN**. **Serum sFlt-1 and VEGF-A levels in patients with FN**. Box plots representing serial concentrations of sFlt-1 and VEGF-A in patients with FN with non-complicated sepsis (n = 31) or septic shock (n = 10) at fever onset and 48 hours thereafter. Mann-Whitney test.

At the time of fever onset no statistical significant difference could be detected between VEGF-A levels in patients with non-complicated sepsis (20.7 pg/ml, range 7.9-129.3 pg/ml) or with septic shock (20.0 pg/ml, range 9.3-158.9 pg/ml; P = 0.9). However, after 48 hours, VEGF-A levels were higher in patients with septic shock (33.0 pg/ml, range 13.0-241.9 pg/ml) compared to patients with non-complicated sepsis (20.9 pg/ml, range 5.6-124.4 pg/ml; P = 0.03) (Figure 1). Similar to VEGF-A, no difference could be observed between sFlt-1 levels at the time of neutropenic fever between patients with non-complicated sepsis (47.3 pg/ml, range 20.8-117.6 pg/ml) and septic shock (49.2 pg/ml, range 29.6-91.1 pg/ml; P = 0.3). However, 48 hours after neutropenic fever a marked difference could be observed between patients with and without septic shock, with increased sFlt-1 concentrations in patients with septic shock (116.0 pg/ml, range 42.7-208.4 pg/ml) compared to patients with non-complicated sepsis (42.9 pg/ml, range 25.9-472.9 pg/ml; P = 0.002) (Figure 2).

### Association of serum sFlt-1 and VEGF-A levels with sepsis prognosis

**Figure 3:**
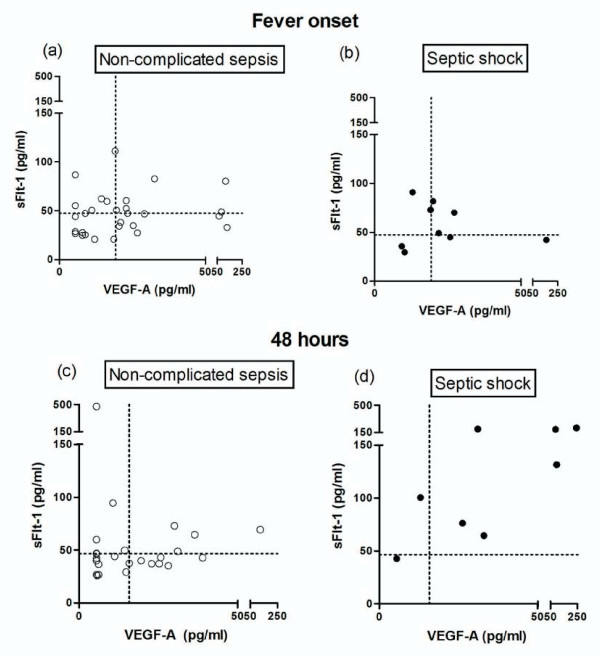
**sFlt-1 and VEGF-A levels in patients with FN**. **Combined analysis sFlt-1 and VEGF-A levels in patients with FN**. Actual sFlt-1 and VEGF-A serum levels obtained at fever onset and after 48 hours are plotted simultaneously as well as median values for each marker at each time point (dotted lines). At fever onset, cases with non-complicated sepsis (empty circles) and septic shock (full circles) are spread evenly across the median values for both parameters (3a-b). After 48 hours, cases that evolved to septic shock seem to localize more frequently in the right upper quadrant (high VEGF-A and high sFlt-1) than cases with non-complicated sepsis, in which levels of both biomarkers change very little.

To explore a potential association of sFlt-1 and VEGF-A levels with sepsis outcome in patients with FN, we first evaluated whether serum VEGF-A and sFlt-1 levels correlated with sepsis severity scores. As shown in Table 2, sFlt-1 measured at fever onset was significantly correlated with both MASCC and SOFA scores. VEGF-A level (measured at fever onset) correlated with SOFA score calculated 48 hours after fever onset (Table 2). Next, we explored whether the individual or combined analysis of sFlt-1 and VEGF-A levels could help in risk stratification of patients with FN. In order to do so, we plotted simultaneously the values of sFlt-1 and VEGF-A in patients with non-complicated sepsis and septic shock, dichotomizing marker levels by their median values. The graphic representation of this analysis seems to indicate that 48 hours after fever onset, patients that evolve to septic shock are more likely to present above median levels of both sFlt-1 and VEGF-A, than patients with non-complicated sepsis (Figure 3). Furthermore, the relative risk for septic shock development in patients with both levels above the median compared to all other patients was 6.9 (1.67-28.5; P = 0.004; Fisher's exact test).

**Table 2:** Correlation of sFlt-1 and VEGF-A with severity of illness

	MASCC	SOFA (Fever onset)	SOFA (48 hours)
**VEGF-A **(fever onset)	Rs = - 0.18 P = 0.31	Rs = - 0.21 P = 0.23	Rs = - 0.17 P = 0.33

**VEGF-A **(48 hours)	**Rs = - 0.43** **P = 0.03**	-	Rs = 0.09 P = 0.66

**sFlt-1 **(Fever onset)	**Rs = - 0.42** **P < 0.01**	**Rs = 0.33** **P = 0.04**	**Rs = 0.32** **P = 0.04**

**sFlt-1 **(48 hours)	Rs = - 0.11 P = 0.52	-	Rs = 0.25 P = 0.16

The Spearman's correlation coefficients (Rs) for sFlt-1 and VEGF-A measured at fever onset and after 48 hours with the severity of illness scores are shown. The correlation of the markers after 48 hours of fever onset with scores at the time of fever onset was not assessed.

Finally, we estimated the diagnostic accuracy of sFlt-1 and VEGF-A levels using ROC procedures in our study population. When measured at fever onset, neither sFlt-1 nor VEGF-A levels yielded area under the ROC curve values that indicated any diagnostic capacity to discriminate patients that would evolve to non-complicated sepsis or to septic shock patients. However, when measured 48 hours after fever onset, when clinical signs of septic shock were still not present in any but one patient, both markers yielded area under the ROC curve values that suggest a diagnostic capacity for the discrimination of FN that evolve to septic shock (Table 3).

**Table 3:** Diagnostic accuracy of sFlt-1 and VEGF-A levels for septic shock development

Biomarker Time-point	sFlt-1	VEGF-A
**Fever onset**		
AUC *	0.61 (0.41-0.81); P = 0.81	0.58 (0.38-0.77); P = 0.47

**48 hours after fever onset**		
AUC *	0.87 (0.73-1.00); **P < 0.01**	0.76 (0.55-0.97); **P = 0.02**

* AUC: area under ROC curve; sFlt-1 and VEGF-A thresholds correspond to the median value for each time point. AUC expressed with 95% confidence interval (CI95%)

## Discussion

Despite improvements in supportive care, complications of sepsis are still one of the main challenges in the management of patients submitted to intensive chemotherapy. Patients with FN are particularly prone to sepsis complications, and because no clinical or laboratory marker can reliably identify patients at lower risk of septic shock, immediate admission and broad-spectrum antibiotics is still the most widely strategy used in the management of patients with FN. Although the use of oral antibiotics in low risk patients has been shown to be safe [15], a robust definition of a low risk patient is still not available [16]. The MASCC score, which is the most studied model, seems to yield, in limited studies, a 71% sensitivity and a 91% positive predictive value to identify low-risk patients [17]. However, subjectivity in clinical assessment of the "disease burden" parameter, the rarity of chronic obstructive lung disease in children and young adults, and its limited validation in the outpatient setting and in patients with acute leukemia still preclude its widespread adoption for the management of FN patients. This opens room for the search of biomarkers that could reliably stratify patients with higher risk of sepsis complications. In addition, preliminary data from animal studies suggest that sFlt-1 could play an important role in the treatment of sepsis, as an endothelial barrier stabilizing agent, provided that the VEGF-A and sFlt-1 axis indeed play clinical relevant roles in the pathogenesis of sepsis complications in humans. Therefore we explored the time-course and the significance of serum levels of sFlt-1 and VEGF-A in patients with FN and hematological malignancies.

The endothelial barrier-breaking properties of VEGF-A are less widely characterized than its mitogenic effects on endothelial cells. Rather than an independent function, this barrier-breaking property is indeed an important part of VEGF-A's role in the regulation of angiogenesis, as the disassemble of an intact endothelial line is necessary for the incorporation of new endothelial cells during vessel sprout. The clinical relevance of this effect in humans was observed more than a decade ago, when patients treated with low dose VEGF-A to boost revascularization in critical limb ischemia presented peripheral edema as a consistent adverse event [18]. Following this observation, elevated VEGF-A levels have been associated with a variety of conditions that share the disruption of the endothelial barrier as a common pathogenic mechanism, including sepsis [19,20]. In intensive care unit patients with sepsis, levels of VEGF-A have also been associated with disease severity and mortality [4-6,21]. More recently, VEGF-A levels were evaluated in a smaller study with patients with FN, which observed higher VEGF-A levels in patients that evolved to severe sepsis compared to patients with non-complicated sepsis [22]. VEGF-A acts by binding to two tyrosine-kinase transmembrane receptors: VEGFR-1 (Flt-1) and VEGFR-2, mainly expressed in endothelial cells. VEGF-A and its receptors act in conjunction with other regulators of angiogenesis such as the angiopoiein/Tie-2 axis, which has also been associated with sepsis diagnosis and outcome by we and others [23-26]. sFlt-1 is a splice variant of the receptor VEGFR-1. sFlt-1 is secreted in soluble form, binds VEGF-A and acts as a decoy receptor, down-regulating its cellular effects. sFlt-1 has been shown to protect mice from VEGF-A induced sepsis [27]. Antagonism of VEGF-A by sFlt-1 has also been explored therapeutically in the treatment of pathogenic vessel growth in cancer and other diseases [28,29]. In our study we demonstrated that patients with FN that evolve to septic shock present higher serum levels of VEGF-A compared to patients with non-complicated sepsis, when measured 48 hours after fever onset. Our observation confirms, in a larger population of patients with septic shock (10 patients), a recent study in patients with FN in which only 1 patient evolved to septic shock [22]. We also describe for the first time that sFlt-1 levels are higher in severely neutropenic patients that evolve to septic shock compared to patients with non-complicated sepsis, and that this increase is only present 48 hours after fever onset. This observation is consistent with recent studies from one group that evaluated sFlt-1 levels in non-neutropenic patients with sepsis and septic shock [6,9] that also observed higher sFlt-1 levels in patients with septic shock, and that sFlt-1 could be a useful biomarker for sepsis severity. Furthermore, we demonstrated that both VEGF-A and sFlt-1 levels correlated with sepsis severity scores.

An interesting finding of our study is the divergent trend of sFlt-1 levels observed in patients that evolve to septic shock (towards higher levels) compared to patients that recover uneventfully (unchanged levels) (Figure 2). This trend seems to indicate that higher sFlt-1 serum levels in the former group of patients could be the expression of an additional compensatory mechanism, triggered by the failure of sFlt-1-independent mechanisms that maintain endothelial barrier in patients of the latter group. In animal models of sepsis, over-expression of sFlt-1 was capable to completely block the barrier-breaking effects of VEGF-A and to reduce mortality, suggesting that sFlt-1 could be used as a regulator of vascular permeability in pathological conditions. However, this was possible by using a gene transfer strategy that resulted in a more than 100-fold increase in sFlt-1 levels [8]. Our data demonstrate that the up to 10-fold elevation of sFlt-1 serum concentration observed in humans was not sufficient to block the development of septic shock in patients with FN. Whether several-fold higher elevations of sFlt-1 could effectively block the endothelial barrier disruption present in patients with septic shock is an exciting scientific question that remains to be answered.

In our study, initial samples were collected very early after fever onset, when no signs of sepsis complications were present. Median time to septic shock development in our study was 4 days, and even samples collected 48 hours after fever were still obtained before the development of overt septic shock, in all but one patient. This was only possible because of the in-hospital design of our study in which patients were under strict monitoring for fever signs, and contrasts with studies of sFlt-1 and VEGF-A levels in non-neutropenic patients, which were mostly performed in intensive care units, after the development of sepsis complications. Even though this specific characteristic of our study does not reproduce real-life practice where a biomarker would be used, it probably allows a more comprehensive evaluation of the kinetics of sFlt-1 and VEGF-A release in human sepsis. In our study, differences in sFlt-1 and VEGF-A levels could not be demonstrated at fever onset, and were only present 48 hours thereafter. This is also in contrast with the observation of higher sFlt-1 and VEGF-A levels in non-neutropenic patients with sepsis at "early" time points. Again, we believe that rather than a difference in the kinetics of sFlt-1 and VEGF-A release in patients with neutropenia, this difference reflects the earlier evaluation of these biomarker levels in our patients compared to previous studies. Indeed, the fact that none of the observed differences in biomarker levels were present at fever onset has important implications. First, it suggests that VEGF-A and sFlt-1 increases are a relatively later consequence of the cascade of events that leads to septic shock. This hypothesis is supported by the intuitive assumption that VEGF-A acts as one of the final downstream elements in the pathogenesis of septic shock, and is consistent with our previous hypothesis that sFlt-1 is released as a salvage compensatory mechanism to restore barrier function. A second clinical implication of our results refers to the use of sFlt-1 and VEGF-A for risk stratification of patients with FN. In our study, the estimation of diagnostic accuracy of VEGF-A and sFlt-1 yielded promising results only when levels were measured 48 hours after fever onset. An ideal biomarker for risk stratification in FN should not require serial sampling, as this would not allow early discharge of low risk patients. Future studies with higher number of patients and under a less controlled environment (including outpatients) are warranted to check whether levels of these biomarkers obtained in a "real world" setting will be able to capture this increase in sFlt-1and/or VEGF-A levels of patients with a worse prognosis and accurately discriminate FN patients with different outcomes.

The major sources of VEGF-A in sepsis are still a matter of debate, and no information on the source of sFlt-1 in sepsis had been published so far. A study with healthy volunteers suggested that platelets, and mainly granulocytes are the sources of more than 90% of circulating VEGF-A [30]. In contrast, in an animal model of sepsis VEGF-A levels increased in liver, kidney and heart, and no difference could be detected between VEGF-A levels in serum and plasma, arguing against a major role of platelets as a source of VEGF-A [8]. In our study, VEGF-A and sFlt-1 serum levels were approximately 5 and 10-fold lower respectively than plasma levels in non-neutropenic septic patients, thus suggesting that platelets and granulocytes do represent an important source of these citokynes in sepsis [6]. However, no difference in neutrophil and platelet counts could be demonstrated between patients with non-complicated sepsis and septic shock at fever onset (Table 1), and no statistical significant correlation could be demonstrated between VEGF-A and sFlt-1 levels with platelet and neutrophil counts at any time-point (data not shown).

Our study has several limitations including a relatively low number of patients, which precludes subgroup analysis, a single-center design and the fact that the in-hospital setting does not reproduce the real-life conditions where a biomarker would be useful. However, our exploratory studied was not aimed to definitively prove or rule out the usefulness of sFlt-1 and VEGF-A determinations as biomarkers of sepsis severity in FN, but rather to test whether these biomarkers showed diagnostic promised under controlled and ideal conditions. In other words, the limitations of our study could also be regarded as its strengths, if it is acknowledged that it was designed to answer a "phase 2 question" in the hierarchy of diagnostic research, setting the stage for a future and planned validating study [31], as well as to gain insights about the time-course of sFlt-1 and VEGF-A release during the very initial phase of sepsis.

## Conclusions

In conclusion, our study demonstrates that patients with hematological malignancies and post-chemotherapy FN that evolve to septic shock present higher levels of sFlt-1 and VEGF-A than patients with non-complicated sepsis, and that levels of these biomarkers correlate with sepsis severity scores. In addition, the time-course of sFlt-1 release suggests that is could represent a salvage compensatory mechanism in patients that evolve to septic shock. Additional studies are warranted to explore the validity of these observations, as well as the feasibility of their incorporation into risk stratification models for neutropenic patients or, in the future, as therapeutic tools in sepsis.

## Competing interests

The authors declare that they have no competing interests.

## Authors' contributions

BEA enrolled patients, recorded clinical data, performed laboratory analysis and contributed to manuscript production; SALM Performed laboratory analysis; FJPA performed statistical analysis and reviewed the manuscript; IL, CADS and JMA contributed to the study design and reviewed the manuscript; EVDP designed the study, analyzed data and contributed to manuscript production. All authors read and approved the final manuscript.
